# Implementation of malaria control programmes during the COVID-19 pandemic in the Southern African Development Community Elimination 8 countries: A scoping review

**DOI:** 10.4102/phcfm.v18i1.5110

**Published:** 2026-02-21

**Authors:** Daphne N. Muzamhindo, Geldine Chironda, Joyce Mahlako Tsoka-Gwegweni

**Affiliations:** 1 Department of Public Health Medicine, University of KwaZulu-Natal, Durban, South Africa; 2 Seed Global Health, Saint John of God University, Mzuzu, Malawi; 3 Department of Public Health, Faculty of Health Sciences, University of the Free State, Bloemfontein, South Africa

**Keywords:** COVID-19 pandemic, malaria control programme, Malaria Elimination 8, programme disruption, SADC, 2030 Elimination Goal

## Abstract

**Background:**

Malaria is one of the communicable diseases affecting the whole world. The World Health Organization (WHO) African Region is the most affected, with the Southern African Development Community (SADC) and the Malaria Elimination 8 (E8) countries accounting for 90% and 95% of the cases, respectively. The WHO tasked the SADC Malaria E8 countries to eliminate malaria by 2030, yet the COVID-19 pandemic response disrupted health programmes.

**Aim:**

The review aims to map and synthesise the evidence on malaria control programmes during the COVID-19 pandemic in the SADC E8 countries to identify gaps, inform policy, enhance planning for future pandemics and promote the attainment of the SADC 2030 Malaria E8 goal.

**Method:**

The reviewers conducted this review using the Joanna Briggs Institute (JBI) methodology. The population, concept and context (PCC) guided inclusion and exclusion criteria. Information relevant to the review questions was extracted using data extraction tools.

**Results:**

Of the 658 articles retrieved, only 7 met the inclusion criteria. Half of the publications were done in 2021, and nothing was published in 2020. The publishers were predominantly public health experts.

**Conclusion:**

There is limited research on the malaria programmes during the COVID-19 pandemic in the Malaria E8 countries.

**Contribution:**

The review brings out the need for research on the topic, policies that promote the continuation of malaria programmes during a pandemic and the employment of coping strategies.

## Introduction

Malaria is one of the first five diseases caused by parasites that affect humans and is one of the main causes of global human deaths in the poor sub-Saharan countries.^1^ The protozoan *Plasmodium* is the causative agent of malaria.^2^ More than 200 million cases and 400 000 deaths are reported each year from 90 countries, and the World Health Organization (WHO) has identified 30 countries for malaria elimination by 2030.^2^ According to the WHO updated malaria terminology 2021, malaria control refers to the efforts made to reduce the malaria disease burden to an extent that can be tolerated in that area.^2^ Based on this definition, malaria control programmes are therefore activities that are implemented to reduce the malaria disease burden in a given area, curb the spread of the disease and achieve the ultimate goal of elimination.

The WHO identified three main focus areas in malaria control, and these are the facts that every affected individual can be prevented, diagnosed and treated for malaria, and that surveillance should be a component of the principal intervention methods.^3^ The WHO’s World Malaria Report 2021 states that programmes are implemented under vector control, case management and surveillance.^4^ According to Zawawi and colleagues, malaria control interventions are concentrated on mass distribution of long-lasting insecticide-treated nets (LLITNs), seasonal malaria chemoprevention, indoor residual spraying (IRS), slide-based diagnosis, rapid diagnostic testing (RDT), case management and raising community awareness.^5^

Programmes have to be consistent to keep abreast of the disease burden. Any interruption of malaria programmes is tantamount to increased case incidence, prevalence and mortality. A survey by the Global Fund in 106 countries revealed that there had been 73% disruptions to malaria control programmes as of June 2020.^6^ The authors further state that a 22% disruption in net distribution and artemisinin-based combined therapy (ACT) would result in cases increasing by 12% and deaths by 35%. Disruption of the same control activities by 75% would result in a 22% increase in cases and twice the mortality, the highest figures in 20 years. Continuation of malaria programmes during pandemics is therefore crucial for the elimination of the disease in malaria-endemic countries.

The coronavirus disease 2019 (COVID-19) pandemic was first reported in December 2019 by the WHO. Coronavirus disease 2019 is caused by the severe acute respiratory syndrome coronavirus 2 (SARS-CoV-2) that is highly virulent and results in high morbidity and mortality. About 101 million COVID-19 cases and 2.4 million deaths were reported in countries that are fighting malaria in the 2 years of the pandemic.^4^ As of June 2021, about 176 million COVID-19 cases and 2.8 million deaths had been reported globally.^7^ The increased morbidity and mortality resulted in the redirection of funds from malaria to the COVID-19 pandemic response.^7^ This resulted in the disruption of malaria programmes, which in turn resulted in increased malaria cases and deaths in malaria-endemic countries worldwide. About 14 million more malaria cases and 47 000 deaths were reported in 2020 than in 2019 globally.^5^ According to the World Malaria Report for 2021, because of the COVID-19 pandemic disruptions in the WHO African Region, from 2019 to 2020, malaria cases went up from 213 million to 228 million; deaths went up from 534 000 to 602 000; case incidence went up from 222 out of 1000 to 233 out of 1000 population at risk and malaria mortality rate rose from 56 out of 100 000 to 62 out of 100 000 population at risk. The WHO African Region is responsible for more than 95% of the malaria cases and 96% of deaths that occurred between 2019 and 2020 because of the COVID-19 pandemic disruptions.^4^

A ‘pulse survey’ report on 65 malaria-endemic countries globally showed that 37 reported that malaria services were partially disrupted (5% – 50%) while 28 had minimum or no disruptions to diagnosis and treatment. During the second round of the survey, 48 malaria-endemic countries responded; 6 of these reported that they had severe disruptions of up to 50%, 15 reported partial disruptions and 27 had no disruptions.^4^ Disruption to vector control programmes included partial or non-distribution of ITNs, delayed IRS and problems with the transportation of resources from suppliers or from distribution points to countries. Disruptions to clinical service (case management) programmes include fewer people reporting to health facilities (HFs) and up to 30% fewer malaria tests carried out in 2020 compared to 2019.^4^ In the WHO African Region, 450 million tests were carried out in 2019, 398 million tests in 2020 and 435 million tests in 2021.^8^

Four countries in the Southern African Development Community (SADC) region were tasked by the WHO to eliminate malaria by 2023, as they had the potential to do so. These countries are Eswatini, Botswana, Namibia and South Africa. However, at an SADC meeting, it was agreed that countries that shared borders with these first-line states join in the elimination initiative for cross-border collaboration in the fight against malaria. The four supporting countries are Angola, Mozambique, Zambia and Zimbabwe. The eight countries (Angola, Botswana Eswatini, Namibia, Mozambique, South Africa, Zambia and Zimbabwe) therefore formed what is called the SADC Malaria Elimination 8 (E8) Initiative. The E8 goal is to drive the malaria elimination agenda in the E8 countries for elimination by 2030.^9^

Successes of the E8 countries include stakeholder engagement, resource mobilisation, keeping malaria on the SADC agenda, establishment of border clinics at entry points, instituting a situation room as a platform for sharing data and emergency preparedness and response plans by member states, training of 118 microscopists and development of a manual for the training of trainers.^9,10^ Such success, to a large extent, promotes implementation of malaria programmes targeting elimination by the year 2030. However, some challenges were experienced, and these include inadequate funding as the E8 countries are no longer eligible for funding because of the economic bracket that they fall into; reluctance by E8 member states to share data and to accept new technology and techniques that promote malaria elimination and incompatibility of malaria budgets with malaria seasonal activities, resulting in delayed procurement and supply of commodities.^9^ These challenges exacerbated the disruption, partial and/or non-implementation of malaria programmes during the COVID-19 pandemic.

### Aim of the review

The aim of this review is to map and synthesise the evidence on the implementation of malaria control programmes during the COVID-19 pandemic in the SADC E8 countries, so as to identify gaps, inform policy and enhance planning for future pandemics and promote attainment of the SADC 2030 Malaria E8 Goal.

### Review questions

#### Overall research question

- *In what ways were malaria control programmes disrupted during the COVID-19 pandemic?*

#### Specific research questions

- *Which malaria control programme activities and policies were implementable during the COVID-19 pandemic?*
- *What is the nature of resources used for malaria control programmes during the COVID-19 pandemic?*
- *What were the barriers to implementation of malaria control programmes during the COVID-19 pandemic?*
- *What coping strategies were used for the continuation of malaria programmes during the COVID-19 pandemic?*

## Methods

This scoping review was conducted in accordance with the JBI methodology for scoping reviews. The methodology includes the inclusion criteria, searching for evidence, selecting the evidence, extracting the evidence, analysing the evidence and presenting the results.^11^

### Inclusion and exclusion criteria

The inclusion and exclusion criteria were determined by population, concept and context (PCC) as shown in Table 1.^12^

**TABLE 1:** Inclusion and exclusion criteria as defined by population, concept and context.

PCC criteria	Inclusion criteria	Exclusion criteria
Population	1. Professional populations involved in malaria control: Environmental health practitioners, environmental health assistants, port health officers (borders), nurses, medical practitioners, laboratory technicians, pharmacists, surveillance officers within the municipalities *Internal stakeholders:* Administrative officers, accounting officers, human resource practitioners *External stakeholders:* NGOs (Global Fund, WHO, CHAI, etc.), municipalities/town councils, government line ministries Non-professional populations involved in malaria control: Labourers, spray operators and communities 2. Participants targeted: Adults (including pregnant women, the elderly and the disabled), infants and children	Populations not involved: Professional and non-professional populations that are not involved in malaria control within the municipalities/government External malaria stakeholder professionals not involved in malaria control Non-professionals from malaria stakeholder institutions and are not involved in malaria control Participants: The mentally disturbed persons
Concept	1. Malaria control programmes; malaria elimination programmes, malaria control initiatives; malaria control strategies, malaria interventions programmes, malaria elimination interventions, malaria prevention programmes, malaria control interventions, malaria reduction, malaria risk and prevention, malaria transmission prevention during the COVID-19 pandemic (2019–2024). 2. COVID-19 pandemic, virus responsible for COVID-19, COVID-19 virus, severe acute respiratory syndrome (SARS) CoV-2 virus, coronavirus, coronavirus disease, 2019 novel coronavirus	1. All malaria control programmes that were conducted prior to the COVID-19 pandemic 2. Non-malaria control programmes that were conducted during the COVID-19 era (i.e. 2019 to date) Any disease or pandemic other than the SARS-CoV-2 virus/COVID-19 pandemic
Context	SADC Malaria Elimination 8 countries: Angola, Botswana, Eswatini, Mozambique, Namibia, South Africa, Zambia and Zimbabwe (Southern Africa)	1. SADC malaria-endemic countries that are not part of the E8: Comoros, Democratic Republic of Congo, Madagascar, Malawi and Tanzania 2. SADC countries certified by WHO to have eliminated malaria: Lesotho, Mauritius and Seychelles

Source: Page MJ, McKenzie JE, Bossuyt PM, et al. The PRISMA 2020 statement: An updated guideline for reporting systematic reviews. BMJ. 2021;372:N71. https://doi.org/10.1136/bmj.n71

PCC, population, concept and context; NGO, non-governmental organisation; WHO, World Health Organization; CHAI, Clinton Health Access Initiative; COVID-19, coronavirus disease 2019; SADC, Southern African Development Community; SARS-CoV-2, severe acute respiratory syndrome coronavirus 2.

### Searching for the evidence

The evidence for this study was searched in selected online academic databases, which are PubMed, Medline, EBSCOhost, Sabinet, ProQuest One Academic, SCOPUS, Web of Science, Google Scholar and Cumulative Index to Nursing and Allied Health Literature (CINAHL).^13^ Any source of information that was relevant to the study was included in the search.^13^ The search for evidence was done in three steps as follows:

*Step 1:* Two online databases that are in line with the topic were first searched. These are EBSCOhost and PubMed. Searching for keywords in the title and abstracts of the sources and of the main terms used in the documents retrieved from this first search was carried out, and the main words used in describing the article were analysed.

*Step 2:* Searching all selected databases using the keywords identified.

*Step 3:* Searching from the list of references of retrieved reports and articles to get more sources.

Where evidence was lacking, then searching was conducted in all retrieved documents including those that were not selected initially. As the review question covers eight countries, primary sources and opinion articles were searched at the same time as the main search from the selected databases. The first two reviewers conduced the search independently, discussed differences and resolved them amicably. The third reviewer verified the searches. The timeframe for the search was 3 months. The search included relevant documents published and unpublished in English. Details of the selected sources with full articles are provided. The Ryan software was used to store the results of the search in preparation for selection.

### Selecting the evidence

Selection of evidence was done in the Ryann systematic review app with the involvement of two reviewers. Prior to the selection of articles, duplicates were removed. The first-level selection was based on the title and abstract. To pilot the selection process, 25 studies were selected based on the title and abstract. The team discussed differences.^15^ There were no disagreements, and hence the third reviewer was not engaged.^16^ The reviewers agreed on 75% of the searches, and the main search commenced in the same manner. The second-level selection was based on full text, with any disagreements being solved along the way. The results were reported in the ScR and presented using the Preferred Reporting Items for Systematic Reviews and Meta-Analysis extension for scoping reviews (PRISMA-ScR).

### Extracting the evidence

A logical and descriptive summary of the results that align with the review questions is presented in the form of a charting table. The key information of the source, such as author, reference and results or findings relevant to the review objectives, is shown. The elements of the PCC are included in the table chart (see Table 2 and Table 3). The tool was adapted from the one provided in the JBI manual and piloted on three sources by two members of the review team to ensure that all the results that are applicable to the search were charted.^15^

**TABLE 2:** Publication characteristics.

Authors (year); Country	Study design	Setting	Professional population involved	Participants targeted
Mbunge et al. (2021); Zimbabwe^17^	Commentary	Malaria endemic areas	Computer scientist	Children and adults
Mbunge et al. (2021); Zimbabwe^17^	Retrospective study	Buhera District Health Facilities	Not stated	Community members
Afai et al. (2021); Mozambique^18^	Descriptive epidemiology	Two health facilities at COVID screening sites	Epidemiologists Community health workers (CHWs)	Patients with malaria symptoms
Roberts et al. (2021); Mozambique^19^	Mixed methods	Communities in malaria foci	Epidemiologists Medical practitioner	Community members Key stakeholders
Moonasar et al. (2023); Namibia^21^	Not stated	SADC	Public health consultant Public health experts Medical researchers	Communities in malaria-endemic districts/provinces
African Union (2023); Angola, Botswana, Eswatini, Mozambique, Namibia, South Africa, Zambia and Zimbabwe^20^	Malaria status report	African countries including Angola, Botswana, Eswatini, Mozambique, Namibia, South Africa, Zambia and Zimbabwe	Not stated	Communities in malaria-endemic districts/provinces
African Union (2023); Zambia^22^	Malaria status report	African countries including Zambia	Not stated	Communities in malaria-endemic districts/provinces
African leaders, Malaria Alliance and RBM Partnership to End malaria (2022); Eswatini^21^		African countries including Eswatini	Not stated	-
African Union Commission, African leaders, Malaria Alliance and RBM Partnership to End malaria (2022); Zambia and Namibia^22^	Malaria status report	African countries including Zambia and Namibia	Not stated	-
African Union Commission, African leaders, Malaria Alliance and RBM Partnership to End malaria (2022); South Africa^22^	Malaria status report	African countries including South Africa	Not stated	Communities in malaria-endemic districts/provinces
African Union Commission, African leaders, Malaria Alliance and RBM Partnership to End malaria (2022); Angola, Namibia, Zambia and Zimbabwe^21^	Malaria status report	African countries including Angola, Namibia, Zambia and Zimbabwe	Not stated	Communities in malaria-endemic districts/provinces
Maharaj (2023), South Africa^24^	Interrupted time series analysis	Limpopo, Mpumalanga and KwaZulu-Natal provinces	Public health experts	Cross-border travellers

Note: Please see the full reference list of the article Muzamhindo DN, Chironda G, Tsoka-Gwegweni JM. Implementation of malaria control programmes during the coronavirus disease 2019 pandemic in the Southern African Development Community Elimination 8 countries: A scoping review. Afr J Prm Health Care Fam Med. 2026;18(1), a5110. https://doi.org/10.4102/phcfm.v18i1.5110 for more information.

COVID-19, coronavirus disease 2019; SADC, Southern African Development Community.

**TABLE 3:** Data extraction table for malaria control programmes, resources and policies used, barriers and coping strategies.

Authors (year); Country	Type of malaria control programmes	Nature of resources and policies	Barriers to implementation	Coping strategies
Mbunge et al. (2021); Zimbabwe^17^	Indoor residual spraying (IRS), LINS distribution, administration of artemisinin-based combination therapy, intermittent preventive treatment (IPT)	Vehicles, human resources, equipment, insecticides, stationery, LINs, medicines, cell phone tablets for data capturing Policies not stated	Movement restrictions, termination of IRS, delayed delivery of IRS chemicals, recursive lockdown	Not stated
Mbunge et al. (2021); Zimbabwe^17^	Case detection Indoor residual spraying	Testing kits Vehicles, human resources, equipment, insecticides, stationery, LINs, medicines Policies not stated	COVID-19 movement restrictions, hampered the recruitment and training of IRS staff, training of the staff and meetings with the community Exposure to COVID-19 through recruitment, trainings, household visits, meetings with stakeholders Risk of contact with contaminated surfaces and objects IRS equipment bought late Scheduled IRS programmes not implemented on time Delays PPE, equipment and chemicals were not delivered on time Recruitment and training of IRS teams was put on hold as the transportation of medical equipment was interrupted, borders were not operational, lockdowns that were ongoing	Not stated
Afai et al. (2021); Mozambique^18^	Malaria testing	RDT kits Antimalarial drugs Policies not stated	Human resources Vehicles Equipment	Use of COVID testing sites to test and treat malaria cases
Roberts et al. (2021); Mozambique^19^	Training IEC campaigns Distribution of IEC materials Malaria prevention and treatment	Laptops, projectors, flip charts, felt pens for training IEC materials, RDT kits Medicines PPEs Vehicles Policies not stated	Not stated specifically for Mozambique Lack of or inadequate PPEs Vehicle maintenance, lack of or inadequate funds for training	Use of new ITNs (PBO nets). This resulted in 50% less cases
Moonasa et al. (2023); Namibia^23^	Reactive focal mass drug administration (rfMDA), or treatment without testing Reactive focal vector control (RAVC) in the form of indoor residual spraying	Vehicles Stationery Policies not stated	Not stated	Not stated
African Union (2023); Angola, Botswana, Eswatini, Mozambique, Namibia, South Africa, Zambia and Zimbabwe^22^	IRS vector control (IRS, larviciding, LLITNs, entomological studies chemoprevention) ITP	Vehicles, HR, equipment, stationary Policies not stated	Limited funds Procurement and supply of commodities, diagnosis, ‘flair ups’, outbreaks in areas where malaria had been eliminated, insecticide and parasite resistance, long walking distances to health facilities Vector control coverages below 95% target in Botswana Mozambique and Zimbabwe (threatens resurgence in susceptible communities). Withdrawal of the global fund in Botswana, Eswatini and Namibia	Not stated
African Union (2023); Zambia^22^	Procurement of commodities	Malaria commodities Policies not stated	Not stated	Allocated additional funds 222% from 2021 to 2023 towards buying items needed to fight malaria
African Union (2023); Eswatini^22^	IRS campaigns Malaria treatment Sensitisation of stakeholders	Financial Fuel Policies not stated	Lack of funds	Use of end malaria fund to pay workers and to buy fuel for IRS campaigns, purchase of antimalarial drugs, redirection of unused COVID-19 funds for malaria programmes ($100 000.00). Use of the funds to sensitise malaria stakeholders on the disease
African Union (2023); Zambia and Namibia^22^	Advocating for malaria in the community Resource mobilisation, mass drug administration campaigns	Vehicles, loud speakers, IEC materials, RDT kits, PPEs, malaria drugs	Not stated	Giving support to community sensitisation programmes, testing, treatment and mass drug administration campaigns
African Union (2021); South Africa^20^	Not stated	Not mentioned. Determined by the programmes	Lack of or inadequate funds	$4 million given to fight malaria in SA Eswatini and Mozambique
African Union (2023); Angola, Namibia, Zambia and Zimbabwe^22^	Cross-border initiatives for information sharing	Financial resources, vehicles	Not mentioned	Not mentioned
Maharaj (2023), South Africa^24^	Malaria testing and treatment	RDT kits, vehicles, medicines	Very few malaria control programmes because staffs were assigned to provide COVID-19 activities	Not mentioned

Note: Please see the full reference list of the article Muzamhindo DN, Chironda G, Tsoka-Gwegweni JM. Implementation of malaria control programmes during the coronavirus disease 2019 pandemic in the Southern African Development Community Elimination 8 countries: A scoping review. Afr J Prm Health Care Fam Med. 2026;18(1), a5110. https://doi.org/10.4102/phcfm.v18i1.5110 for more information.

COVID-19, coronavirus disease 2019; PPE, personal protective equipment; IRS, indoor residual spraying; LLITN, long-lasting insecticide-treated nets; IEC, information, education and communication; RDT, rapid diagnostic testing; LLINS, long lasting insecticidal nets; PBO, piperonyl butoxide; RBM, roll back malaria; HR, human resources; ITP, intermittent preventive treatment; ITN, insecticide-treated net.

### Analysis and presentation of the evidence

The evidence was analysed by counting, identifying and describing concepts, programmes and other criteria of data collected, as these would help meet the objective of the review.^14^ The evidence was presented using tables, pie charts and graphs.^15^ The number of times that a concept appeared, the population and the place where the study was conducted were also considered.^16^

## Results

**FIGURE 1:**
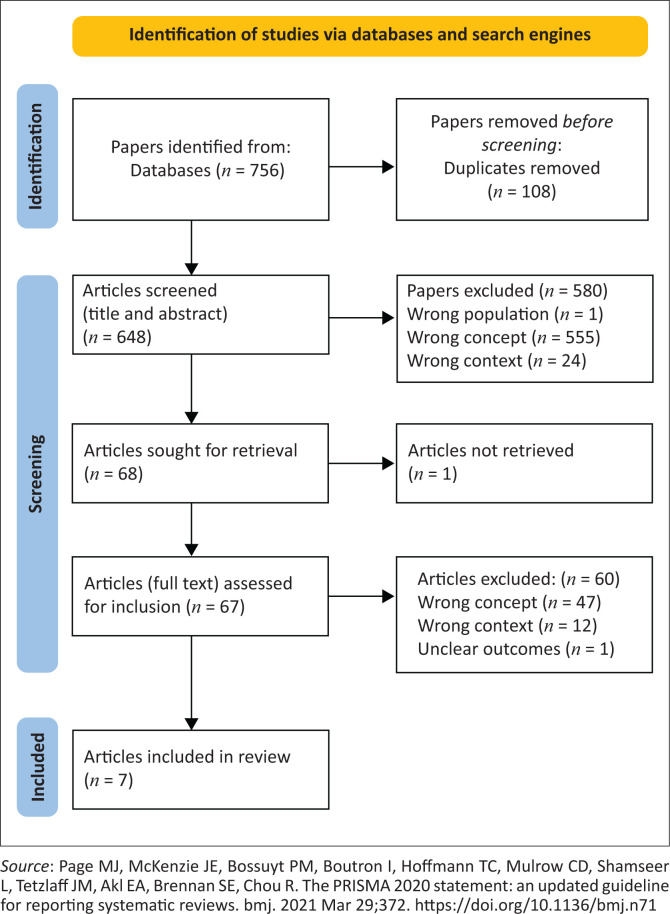
Preferred reporting items for systematic reviews and meta-analyses.

A total of 756 articles were selected from five databases. Duplicates identified and removed were 108. The second-level selection, which was based on the title and abstract, yielded 648 articles. The reasons for the exclusion of articles were wrong context (*n* = 24), wrong concept (*n* = 555) and wrong population (*n* = 1). The final selection was based on full-text screening, and four articles were selected out of 67. Two other articles were identified from grey literature. The review is based on the seven final articles selected, as shown in Figure 1.

### Publication characteristics

**FIGURE 2:**
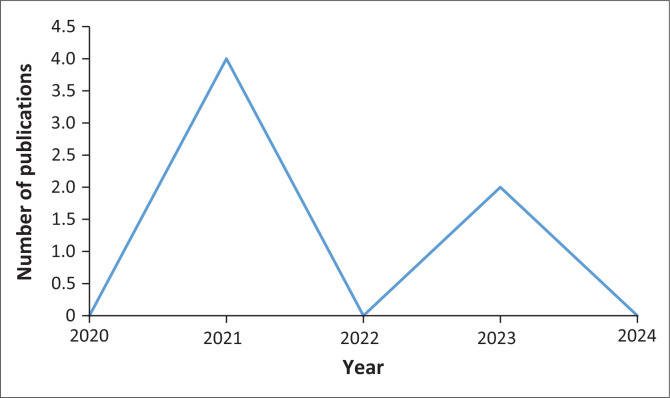
Number of publications on malaria programmes during the COVID-19 pandemic in the Southern African Development Community Malaria E8 countries (*n* = 7).

All the articles selected were written in English. Publication dates ranged from 2020 to 2023. More than half of the publications were published in 2021 (*n* = 4), followed by 2023 (*n* = 2). The year 2022 had the least publications (*n* = 1). Nothing was published in 2020 (*n* = 0). Five (*n* = 5) articles were selected from journals, while two (*n* = 2) were selected from grey literature (see Figure 2).

**FIGURE 3:**
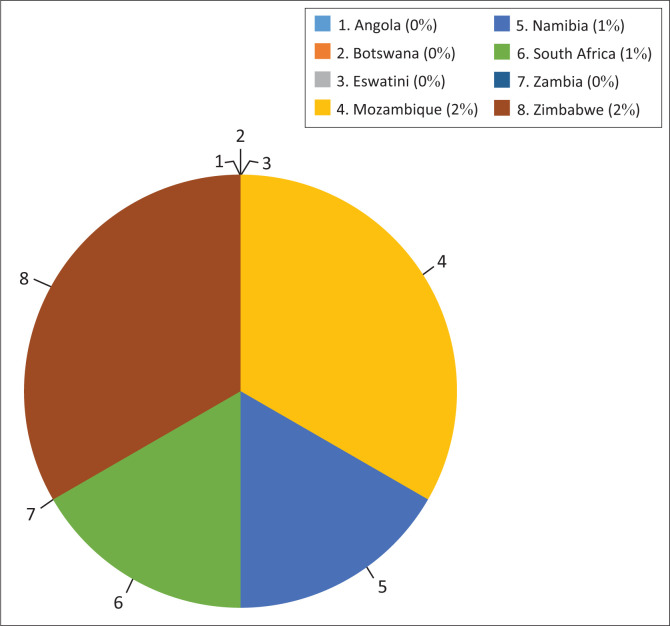
Number of publications during the COVID-19 pandemic, per E8 country.

**FIGURE 4:**
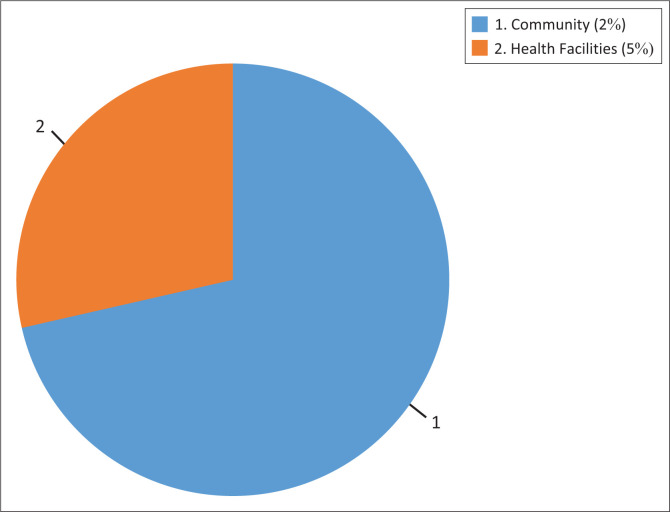
Study settings for the publications on malaria programmes in the Southern African Development Community Malaria E8 countries (*n* = 7).

Of the Malaria Elimination 8 countries, publications were available for half of the countries, which are Mozambique, Namibia, South Africa and Zimbabwe. Among these four, the countries with the most publications were Mozambique (*n* = 2) and Zimbabwe (*n* = 2). Namibia and South Africa had one publication each (*n* = 1) (Figure 4). The remaining E8 countries (Angola, Botswana, Eswatini and Zambia) were mentioned in grey literature, but there was no publication specific to the countries (Table 2; see Figure 3).

There was no dominant study design, as each article had a different study design. The designs included a retrospective study (*n*-1), descriptive epidemiology (*n* = 1), mixed methods (*n* = 1) and interrupted time series analysis. There was one commentary (*n* = 1) and one report (*n* = 1). In one case (*n* = 1), there was no mention of the study design. The professional populations involved in the studies included computer scientist (*n* = 1), epidemiologists, community health workers (CHWs), medical practitioners, public health experts and medical researchers. Community members (children and adults) in malaria-endemic districts and provinces, patients with malaria symptoms, cross-border travellers and key stakeholders were the participants targeted for the studies (see Table 2).

### Activities of malaria control programmes during the COVID-19 pandemic

The malaria control programme activities that were implemented in all the E8 countries include the dissemination of information to sensitise the community and other stakeholders and cross-border travellers about malaria, vector control, case management, resource mobilisation and trainings. The activities conducted to disseminate the information on malaria and to advocate for the programmes were holding campaigns to sensitise communities and other stakeholders on malaria disease and distribution of Information, Education and Communication (IEC) materials. Such activities were implemented in Eswatini and Zambia. The vector control activities that were conducted were IRS, larviciding and distribution of LLTINs. Entomological studies were carried out in all the E8 countries. Case management activities include case detection through testing, administering artemisinin-based combination therapy, intermittent preventive treatment (IPT), reactive focal mass drug administration (rfMDA) or treatment without testing and related campaigns treating malaria cases. Testing and treating activities were conducted in all the E8 countries, while rfMDA was only carried out in Zambia. Resource mobilisation and the procurement of commodities required to implement prevention and control activities were carried out only in Zambia. Training on prevention and control activities was conducted only in Mozambique. Zimbabwe is the only country that implemented cross-border activities (see Table 4).

**TABLE 4:** Implementation of malaria programme activities per country during the COVID-19 pandemic in the Southern African Development Community Malaria Elimination 8 countries.

Country	Dissemination of information	Indoor residual spraying	Insecticide-treated net distribution	Larviciding	Entomological studies	Reactive focal mass drug administration (rfMDA)	Test and treat	Resource mobilisation	Procurement of commodities	Trainings	Cross-border activities
Angola	-	X	X	X	X	-	x	-	-	-	-
Botswana	-	X	X	X	X	-	x	-	-	-	-
Eswatini	X	X	X	X	X	-	x	-	-	-	-
Mozambique	-	X	X	X	X	-	x	-	-	X	-
Namibia	-	X	X	X	X	-	x	-	-	-	-
South Africa	-	X	X	X	X	-	x	-	-	-	-
Zambia	X	X	X	X	X	x	x	x	x	-	-
Zimbabwe	-	X	X	X	X	-	x	-	-	-	x

### Resources

Human and financial resources, vehicles, fuel, equipment, IEC materials, stationary and malaria commodities cut across the board all activities. Resources become activity specific. These were rapid diagnostic and treatment kits for malaria testing; medicines or antimalarial drugs for treatment of cases; insecticides for spraying structures; LLINs for preventing vector–human contact; personal protective equipment (PPEs); stationery for records; electronic devices like cell phones and tablets for data capturing and laptops, projectors and flip charts for training.

### Barriers to implementation

While the different countries had common barriers, some were unique for a particular country. Movement restrictions, lack of or inadequate funds, non-supply of commodities, outbreaks where malaria had been eliminated, insecticide and parasite resistance and long walking distances to HFs were the barriers that were reported across the board. Zimbabwe reported the greatest number and kinds of barriers, while Angola, Botswana, Eswatini and South Africa reported fewer barriers besides those that were cross-cutting. Barriers that were reported in Zimbabwe but not in other countries were non-recruitment and training of staff, exposure to the COVID-19 virus through community engagements that would expose staff to contaminated surfaces, household visits and meetings with stakeholders. The reassignment of staff to respond to the COVID-19 pandemic was reported in South Africa. Staff shortage was reported in Mozambique. Insecticide and parasite resistance were not captured in the articles, but they were mentioned in the grey literature. Spray coverages of less than 95% were reported in Botswana, Mozambique and Zimbabwe. Botswana, Eswatini and Namibia reported the withdrawal of the Global Fund (Table 5).

**TABLE 5:** Barriers to the implementation of malaria programmes.

Barriers	Movement restrictions	Inadequate funds	Non-supply of commodities	Outbreaks where malaria had been eliminated	Insecticide resistance	Parasite resistance	Long walking distances to health facilities	Withdrawal of global fund	Human resource shortage/reassignment	Termination/delayed IRS	Non-recruitment of IRS staff	Late procurement of IRS equipment	Delayed delivery of PPE and equipment	Recruitment and training of staff and teams put on hold	Shortage of vehicles	Vehicles not maintained
Angola	-	x	X	x	X	x	X	-	-	-	-	x	-	-	-	-
Botswana	-	x	X	x	X	x	X	X	-	-	-	x	-	-	-	-
Eswatini	-	x	X	x	X	x	X	X	-	-	-	x	-	-	-	-
Mozambique	-	x	X	x	X	x	X	-	x	-	-	x	x	-	X	x
Namibia	-	x	X	x	X	x	X	X	-	-	-	x	-	-	-	-
South Africa	-	x	X	x	X	x	X	-	x	-	-	x	-	-	-	-
Zambia	-	-	-	-	-	-	-	-	-	-	-	x	-	-	X	-
Zimbabwe	-	x	X	x	X	x	X	-	-	X	x	x	x	x	X	-

PPE, personal protective equipment; IRS, indoor residual spraying.

### Coping strategies

The coping strategies used were in the form of infrastructure (testing sites), commodities (PBO [piperonyl butoxide] mosquito nets) and additional monetary resources. Monetary resources were the most used coping strategy reported, followed by commodities. In Zambia, additional funds (222%) from 2021 to 2023 were availed towards buying items needed to fight malaria. In Eswatini, the End Malaria Fund was used to pay workers, to buy fuel for IRS campaigns and to purchase antimalarial drugs. In addition, unused COVID-19 funds were redirected for use in malaria programmes ($100 000.00). The funds were used to sensitise malaria stakeholders on malaria. Infrastructure was mentioned in one instance. In Mozambique, the coping strategy was in the form of infrastructure and commodities. Coronavirus disease 2019 testing sites were used to test and treat malaria cases, and new ITNs (PBO nets) were used for vector control, resulting in the reduction of cases by 50%. In Zambia and Namibia, support was given to malaria community sensitisation programmes testing, treatment and mass drug administration campaigns. However, the literature was silent on the nature of the support being referred to. South Africa, Eswatini and Mozambique received $4 million to fight malaria. No coping strategies were mentioned for Angola, Botswana and Zimbabwe (Table 6).

**TABLE 6:** Coping strategies used in malaria programmes in the different E8 countries during the COVID-19 pandemic.

Country	Coping strategy
Angola	Not mentioned
Botswana	Not mentioned
Eswatini	Funding: COVID-19 funds redirected for use in malaria programmes ($100 000.00)
Mozambique	Infrastructure and commodities (PBO nets, COVID testing kits to test malaria)
Namibia	Not stated
South Africa	Funding
Zambia	Funding towards buying items needed to fight malaria
Zimbabwe	Not mentioned

COVID-19, coronavirus disease 2019; PBO, piperonyl butoxide.

## Discussion

The sources of literature answered the review questions that sought to identify the programmes that were implemented, the types of resources used, the barriers to the implementation of programmes and the coping strategies employed during the COVID-19 pandemic. All publications included in this review were published during the COVID-19 pandemic. Probably that is the reason why they are so few, given the disruptions and movement limitations during that time. Although there were few publications, most were done in 2021. This could be attributed to the fact that researchers were beginning to understand the novel disease. Ironically, the lack of publications in 2022, when researchers were becoming more accustomed to the COVID-19 disease, could indicate lack of knowledge among researchers about the E8 agenda. It is highly unlikely that researchers could remain silent if they were aware of the agenda and the malaria disease burden in the E8 countries, let alone its implication for the regional and global malaria disease burden.

Although more than half of the publications were on Zimbabwe, half of those were published by the same author, which is why there were more publications on Zimbabwe. These two translate to a quarter of the total publications in the E8 countries, during the period under review. In terms of publishers, therefore, Mozambique then becomes the country with the most contributors. The lack of publishers in Angola, Botswana, Eswatini and Zambia could indicate a lack of knowledge about the E8 Malaria Elimination Agenda and the 2030 elimination goal, a lack of funds to conduct the research or a lack of interest or motivation.

According to an educational article by Peters et al., a lot of euros was set aside for research during the COVID-19 pandemic, and researchers conducted lots of research, of which 10% – 20% were biomedical issues.^25^ The writer further states that 20 000 papers were published from the time the pandemic started, and this resulted in the sharing of information from some papers before peer review was done. The notion that research on the review topic was possibly not conducted because of the unavailability of funds could, therefore, be refuted. Global and regional malaria stakeholders could not have missed the information on the availability of research funds. On the contrary, the writer submits that there was a lack of research funding from public funds and that funds were redirected to the COVID-19 response. This move explains why there was little or no research on malaria programmes in the E8 countries during COVID-19. Peters et al. state that research is crucial, as it helps prepare for future pandemics and informs the response.^26^

According to a commentary by Lamarre and colleagues, there were fewer women researchers in March 2020, with the assumption that women would be at home taking care of responsibilities, hence the increase in male researchers during that time. The writers further state that the lack of funds discourages and prevents first-time authors from conducting research, thereby depriving the world of important information that would otherwise benefit the world.^17^ Global and regional malaria stakeholders should lobby for funds for research from donors and have them channelled to SADC Malaria Elimination research to promote the attainment of the 2030 elimination goal.

### Strength and limitations

This review explores malaria programmes that were implemented in the E8 countries during the COVID-19 pandemic, which is crucial as the information from the study would inform malaria programmes so as to support the attainment of the 2030 Malaria E8 goal. Secondly, the review revealed the gaps in knowledge and research in some of the E8 countries. Thirdly, a total of two reviewers conducted the search and selection of articles, which eliminates any form of bias. Where opinions differed, discussions took place and differences were resolved amicably; therefore, the third reviewer did not have to be engaged to resolve the differences.

Only literature published in English was considered for this review. Both published and grey literature on the topic under review were generally scanty. Published literature for Zimbabwe was published by one publisher, limiting the views of other prospective reviewers. Although there was information on Angola, Botswana, Eswatini and Zambia, the lack of publications on these countries does not give a comprehensive picture of the topic under review, pertaining to these individual countries. Since the review was the first one, no literature was found to compare and contrast the results with.

### Recommendations for practice

Funding was one of the major hindrances to the continuation of malaria programmes during the COVID-19 pandemic. The SADC Malaria E8 countries are all member states of the African Union (AU). Harper and colleagues reported that the high malaria disease burden in Africa is caused by poverty, low tax revenues, heavy debts, financial crisis, inadequate local resources and high costs of borrowing funds.^27^ This necessitates the need for funding. The report recommends that $1.5 billion be made available to enable the implementation of current programmes in the member states during the period 2024–2026 in view of the financial crisis worldwide, increased transportation costs for commodities, insecticide resistance and ‘partial drug resistance’.

The report further advocates for $5 billion each year, so that progress is made towards elimination and the allocation of more local funds (funds to be increased by $300 million among member states). Currently, 70% of malaria resources are funded, indicating that member states rely on donors. According to the same report, Zambia increased domestic funding by 174% and malaria commodities by 222% from 2021 to 2023. If all member states followed suit, the malaria disease burden would be under control such that, even during a pandemic, malaria programmes would not be significantly affected. H.E. President Umarco Sossoco Embalo recommends that funds be availed from many sectors, including high-level national funds to end malaria, prioritising health and funding of malaria programmes, international funding and the use of malaria as a ‘pathfinder’ to reinforce health systems, to be ready for pandemics and climate change and to mitigate and adapt health programmes.^28^

Malaria elimination should not be solely a state problem. There needs to be a national paradigm shift in which the E8 nations are sensitised to appreciate the fact that malaria elimination is not solely a government issue but the responsibility of every sector and individual. Governments of the E8 countries should have policies that engage all sectors and workers to become malaria stakeholders for elimination of the disease as the health sector budget alone would never manage. Each sector should budget for and set aside, from its budget allocation, a certain percentage towards the national malaria contingency fund. All workers should be mobilised and politicised to appreciate and accept the fact that they have a role to play in the elimination of malaria and the attainment of the 2030 elimination goal. Each worker should pay a malaria tax towards the malaria contingency fund, based on income, but the amount should not exceed $1.00 per month, so that in the event of a pandemic or disaster, malaria control programmes will continue without any interruption. Massive campaigns would have to be conducted on mainstream and social media to educate the nation in the E8 countries.

The barrier of a lack of human resources could be resolved by capacitating communities to be able to deal with malaria cases during a pandemic or disaster. More CHWs should be recruited and trained to serve their immediate communities at the village level, such that in cases of pandemics such as COVID-19, where there are movement restrictions, communities will be able to respond to malaria with the help of CHWs. The AU Report 2021 advocates for the recruitment and training of CHWs for community advocacy and case management, particularly in areas that are not easily reached, and for strengthening response mechanisms for pandemic preparedness and response.^21,22^ The use of scorecards where communities record information affecting health service delivery has been used successfully in Zambia. Community needs, experiences and gaps are reported directly to the relevant offices and are addressed in a timely manner. This practice is recommended for the E8 countries, as first-hand information would result in appropriate and effective responses during a pandemic, thereby promoting the continuation of programmes during a pandemic and system resilience, as control would be directed to the high-burden areas.

The non-supply of commodities could be resolved through the local manufacture of malaria commodities, thereby fostering system resilience and economic development, as well as promoting increased access to such commodities. The Kingdom of Eswatini envisages to come up with a factory that packages malaria commodities so as to raise funds for local malaria control programmes.^29^ The report further recommended the ratification of the African Medicines Treaty for implementation in 2022 and that barriers impeding the local manufacture of malaria commodities be addressed by the African Continental Free Trade Agreement framework, regional economic communities and national regulators.

Parasite resistance results in the clearing of parasites in malaria patients taking longer, and medication will not be effective in such a patient. Parasite resistance can be resolved through entomological studies and surveillance to detect and monitor resistance. Research on resistance in each Malaria E8 country should be conducted to inform malaria control initiatives. The WHO recommends that there be improved resistance detection and prevention of the spread of resistant parasites.^27^ Insecticide resistance has been resolved through the use of PBO nets, which have been proved effective. In Mozambique, cases decreased by about 50% when PBO nets were used. The use of next-generation ITNs is reported to be twice more effective. In the same vein, the use of next-generation chemicals is recommended for insecticide resistance, which also helps deal with parasite resistance.^28^

The use of CHWs to attend to malaria programmes at the community level could go a long way towards addressing the problem of patients having to walk long distances to access treatment at HFs.^27^ Trained CHWs should be capacitated and authorised to attend to malaria patients in the community during a pandemic. Community workers should also be capacitated to detect and report malaria cases to CHWs so that they are attended to in a timely manner at the community level, during a pandemic.

Training in any given health initiative, as well as in other setups, is vital for capacitating implementers. It also enhances effectiveness, efficiency and excellence in service provision. However, the COVID-19 pandemic taught us that recruitment and training may not be possible. It is therefore paramount that malaria programme managers at national and sub-national levels be proactive and ensure that communities are capacitated and equipped to conduct IRS and larviciding in their own neighbourhoods and homes. Communities should be urged to use the mosquito nets from previous years to prevent the spread of the disease through the distribution of ITNs during a pandemic.

### Recommendations for research and policy

The Malaria E8 agenda should be well shared within academia and among stakeholders so that it is supported from all quarters through research, funding, donations and other means. There is a need for SADC to fund or source funds for research on the malaria initiative in each of the E8 countries so that gaps that could hinder the attainment of the 2030 goal are identified and dealt with timeously. Researchers should be directed towards researching on countries that lack information on the malaria agenda, for example, Angola, Eswatini and Zambia. Malaria managers and public health students in higher institutions of learning should be encouraged to research malaria-related issues affecting the attainment of the SADC E8 goal so that there is authentic information as much as possible for effective decision-making, thereby maintaining the trajectory towards attainment of the 2030 elimination goal, even during a pandemic.

Harper et al. define policies as ‘laws, regulations, judicial decrees, agency guidelines and budget priorities’.^27^ Policies are informed by research, as strong evidence is required before change of policy or formulation of a new one. Furthermore, there is a need for proactivity in research so that, when the need for a new policy or policy change arises, evidence from research is already available to inform the policies accordingly.^28^ These notions suggest that the absence of policies that promote the continuation of malaria programmes during the COVID-19 pandemic could be the result of a lack of authentic information from research to inform policy, as evidenced by lack of research articles on the topic. In the United States, $30 billion is used on health research every year to promote public health.^26^

The need for policies that are peculiar to the Malaria Elimination agenda in the E8 countries to be identified and developed for implementation, for the attainment of the 2030 elimination goal, for example, a policy for the continuation of malaria programmes during a pandemic or disaster in the E8 countries. Stakeholder reports, for example, WHO and the African Union, should specifically address malaria control in the E8 countries, given the fact that the countries are responsible for 90% of the global disease burden. The attainment of the 2030 malaria elimination goal in the E8 countries would therefore go a long way towards the regional and global elimination of malaria.

Policies peculiar to a pandemic should be synchronised with existing policies for the elimination of malaria so that they complement each other instead of being retrogressive towards the attainment of the respective elimination targets; hence, one disease will not be contained at the expense of the other, as happened during the COVID-19 pandemic – resources to fight malaria were redirected to respond to the COVID-19 pandemic.

## Conclusion

The findings of this review indicate that, in general, research on malaria programmes in the E8 countries during the COVID-19 pandemic was scant, even though there is a large professional population in relevant fields. This is detrimental to the achievement of the SADC E8 goal because the drivers of the agenda will lack information affecting the initiative that promotes attainment of the 2030 goal. Malaria managers and public health researchers should be encouraged to conduct research that supports this agenda in the E8 countries, even during a pandemic or disaster, until the goal is attained. The initiative should be shared with the public to gain the support of public health researchers, all sectors, communities and workers. International and national malaria stakeholders and the respective national governments should lobby for funds to be used for research in the Malaria E8 countries. The funds are to be earmarked for the continuation of malaria programmes during a pandemic and, consequently, the attainment of the 2030 elimination goal.

National governments should take the lead in malaria control initiatives, instead of relying on external donors for support. Advocacy and mobilisation of funds for the research should therefore be scaled up in preparation for a pandemic or disaster. Because malaria elimination is in the interest of all citizens, fundraising could take the form of an Emergency Fund that is collected through a minimal malaria tax on all salaried employees and levies on registered private companies. Partial and/or non-implementation of malaria control programmes is retrogressive for attainment of the elimination, as coverage targets will not be met. The financial barrier has ripple effects on the implementation of all prevention and control programmes that require finances. This gap should be addressed for the benefit of malaria control initiatives during a pandemic. The local manufacture of malaria commodities would go a long way to ensure consistent supply as well as improve access for the continuation of malaria programmes during a pandemic. The coping strategies employed during the COVID-19 pandemic could not match the demand. Policies that engage all sectors, communities and workers as drivers of the elimination agenda would enhance system resilience during a pandemic. Putting these in place will ensure the continuation of malaria programmes during future pandemics.

## References

[1] Oyegoke OO, Maharaj L, Akoniyon OP, et al. Malaria diagnostic methods with the elimination goal in view. Parasitol Res. 2022;121(7):1867–1885. 10.1007/s00436-022-07512-9 35460369 PMC9033523

[2] Oyegoke OO, Adewumi TS, Aderoju SA, et al. Towards malaria elimination: Analysis of travel history and case forecasting using the SARIMA model in Limpopo Province. Parasitol Res. 2023;122:1775–1785. 10.1007/s00436-023-07870-y 37310511 PMC10261840

[3] Lindblade KA, Li XH, Tiffany A, et al. Supporting countries to achieve their malaria elimination goals: The WHO E-2020 initiative. Malar J. 2021;20(1):1–11. 10.1186/s12936-021-03998-3 34930239 PMC8686104

[4] WHO. WHO malaria terminology WHO global malaria fund [homepage on the Internet] 2021. [cited 2025 Nov 25]. Available from: https://www.who.int/publications/i/item/9789240038400

[5] Zawawi A, Alghanmi M, Alsaady I, Gattan H, Zakai H, Couper K. The impact of COVID-19 pandemic on malaria elimination. Parasite Epidemiol Control. 2020;11:e00187. 10.1016/j.parepi.2020.e00187 33102823 PMC7574840

[6] Chipukuma HM, Halwiindi H, Zulu JM, Azizi SC, Jacobs C. Evaluating fidelity of community health worker roles in malaria prevention and control programs in Livingstone District, Zambia – A bottleneck analysis. BMC Health Serv Res. 2020;20(1):612. 10.1186/s12913-020-05458-1 32615960 PMC7331272

[7] SADC. SADC annual report 2020/2021 [homepage on the Internet]. SADC; 2020. [cited 2025 Nov 10]. Available from: https://www.sadc.int/document/sadc-annual-report-2020-2021

[8] Inzaule SC, Ondoa P, Loembe MM, Yenew KT, Ogwell OAE, Nkengasong JN. COVID-19 and indirect health implications in Africa: Impact, mitigation measures, and lessons learned for improved disease control. PLoS Med. 2021;18(6):e1003666. 10.1371/journal.pmed.1003666 34161318 PMC8266084

[9] Anna-Katharina H, Lu G, Razum O, et al. Public health-relevant consequences of the COVID-19 pandemic on malaria in sub-Saharan Africa: A scoping review. Malar J. 2021;20:1–16. 10.1186/s12936-021-03872-2 34380494 PMC8355579

[10] Chen JH, Fen J, Zhou XN. From 30 million to zero malaria cases in China: Lessons learned for China–Africa collaboration in malaria elimination. Infect Dis Poverty. 2021;10(1):51. 10.1186/s40249-021-00839-y 33875017 PMC8055304

[11] WHO. World malaria report 2022. Geneva: WHO; 2022.

[12] Page MJ, McKenzie JE, Bossuyt PM, et al. The PRISMA 2020 statement: An updated guideline for reporting systematic reviews. BMJ. 2021;372:N71. 10.1136/bmj.n71 33782057 PMC8005924

[13] Raman J, Fakudze P, Sikaala CH, Chimumbwa J, Moonasar D. Eliminating malaria from the margins of transmission in Southern Africa through the elimination 8 initiative. Trans R Soc S Afr. 2021;76(2):137–145. 10.1080/0035919X.2021.1915410

[14] SADC. SADC annual report 2020/2021. Gaborone: SADC; 2020.

[15] Peters MD, Godfrey C, McInerney P, Munn Z, Tricco AC, Khalil H. Chapter 11: Scoping reviews. JBI Evid Synth. 2020;169(7):467–473. 10.46658/JBIRM-20-01 33038124

[16] Kinney MV, Walugembe DR, Wanduru P, Waiswa P, George A. Maternal and perinatal death surveillance and response in low-and middle-income countries: A scoping review of implementation factors. Health Policy Plan. 2021;36(6):955–973. 10.1093/heapol/czab011 33712840 PMC8227470

[17] Mbunge E, Millham R, Sibiya N, Takavarasha Jr S. Is malaria elimination a distant dream? Reconsidering malaria elimination strategies in Zimbabwe. Public Health in Practice. 2021;2:100168. 34514451 10.1016/j.puhip.2021.100168 PMC8417459

[18] Afai G, Banze AR, Candrinho B, Baltazar CS, Rossetto EV. Challenges for malaria surveillance during the COVID-19 emergency response in Nampula, Mozambique, January–May 2020. Pan African Medical Journal. 2021;38(1). 10.11604/pamj.2021.38.254.27481 PMC8164433 34104302

[19] Roberts KW, Smith Gueye C, Baltzell K, Ntuku H, McCreesh P, Maglior A, et al. Community acceptance of reactive focal mass drug administration and reactive focal vector control using indoor residual spraying: A mixed-methods study in Zambezi region, Namibia. Malaria Journal. 2021;20:1–11. 33752673 10.1186/s12936-021-03679-1 PMC7986500

[20] African Union. African union malaria progress report 2021. Addis Ababa: African Union; 2021.

[21] African leaders call for immediate action to save additional lives from malaria in the face of COVID-19 [press release]. AllAfrica.com. 2020 May 11.

[22] African Union. African union malaria status report 2022. Addis Ababa: African Union; 2023.

[23] Moonasar D, Chimumbwa J, Leonard E, Murugasampillay S, Maharaj R. Malaria control and elimination in southern Africa – progress, challenges, and priorities. J Communicable Dis. 2023;56–59.

[24] Maharaj R, Ward A, Bradley D, Seocharan I, Firas N, Balawanth R, et al. The effect of the COVID-19 lockdown on malaria transmission in South Africa. Malaria Journal. 2023;22:1–8. 36964548 10.1186/s12936-023-04542-1 PMC10038361

[25] Arksey H, O’Malley L. Scoping studies: Towards a methodological framework. Int J Soc Res Methodol. 2005;8(1):19–32. 10.1080/1364557032000119616

[26] Peters MD, Godfrey C, McInerney P, et al. Best practice guidance and reporting items for the development of scoping review protocols. JBI Evid Synth. 2022;20(4):953–968. 10.11124/JBIES-21-00242 35102103

[27] Sharma P, Goyal N. How to write a scoping review? Int J Adv Med Health Res. 2023;10(1):53–56. 10.4103/ijamr.ijamr_91_23

[28] Harper L, Kalfa N, Beckers GMA, et al. The impact of COVID-19 on research. J Paediatr Urol. 2020;16(5):715–716. 10.1016/j.jpurol.2020.07.002 PMC7343645 32713792

[29] Brownson RC, Chriqui JF, Stamatakis KA. Policy, politics, and collective action. Am J Public Health. 2009;99(9):1576–1583. 10.2105/AJPH.2008.156224 19608941 PMC2724448

